# Plantar Temperature Response to Walking in Diabetes with and without Acute Charcot: The Charcot Activity Response Test

**DOI:** 10.1155/2012/140968

**Published:** 2012-07-30

**Authors:** Bijan Najafi, James S. Wrobel, Gurtej Grewal, Robert A. Menzies, Talal K. Talal, Mahmoud Zirie, David G. Armstrong

**Affiliations:** ^1^Southern Arizona Limb Salvage Alliance (SALSA), University of Arizona College of Medicine, Tucson, AZ, USA; ^2^Arizona Center on Aging, University of Arizona, Tucson, AZ, USA; ^3^University of Michigan, Medical School, Ann Arbor, MI, USA; ^4^Diabetic Foot and Wound Center, Department of Medicine, Hamad Medical Corporation, Doha, Qatar

## Abstract

*Objective*. Asymmetric plantar temperature differences secondary to inflammation is a hallmark for the diagnosis and treatment response of Charcot foot syndrome. However, little attention has been given to temperature response to activity. We examined dynamic changes in plantar temperature (PT) as a function of graduated walking activity to quantify thermal responses during the first 200 steps. *Methods*. Fifteen individuals with Acute Charcot neuroarthropathy (CN) and 17 non-CN participants with type 2 diabetes and peripheral neuropathy were recruited. All participants walked for two predefined paths of 50 and 150 steps. A thermal image was acquired at baseline after acclimatization and immediately after each walking trial. The PT response as a function of number of steps was examined using a validated wearable sensor technology. The hot spot temperature was identified by the 95th percentile of measured temperature at each anatomical region (hind/mid/forefoot). *Results*. During initial activity, the PT was reduced in all participants, but the temperature drop for the nonaffected foot was 1.9 times greater than the affected side in CN group (*P* = 0.04). Interestingly, the PT in CN was sharply increased after 50 steps for both feet, while no difference was observed in non-CN between 50 and 200 steps. *Conclusions*. The variability in thermal response to the graduated walking activity between Charcot and non-Charcot feet warrants future investigation to provide further insight into the correlation between thermal response and ulcer/Charcot development. This stress test may be helpful to differentiate CN and its response to treatment earlier in its course.

## 1. Background

Charcot neuroarthropathy (CN) is a devastating complication of diabetes. It has a similar mortality rate as lower extremity ulceration and a twofold higher rate of major amputation compared to those without CN [1]. It has been estimated that 63% of CN patients will develop a foot ulcer [2]. The combination of foot ulcer and CN increases the risk of amputation 12-fold [3]. The increased mortality risk associated with CN appears to be independent of foot ulcer and other comorbidities [2].

What further complicates CN is that there is no clear definition for it [4]. There are no pathologic markers or diagnostic criteria. Therefore, the diagnosis relies on pattern recognition and clinical intuition [5]. Not surprisingly, the diagnosis can be missed up to 95% of the time [6] and the average diagnostic delay has been estimated at almost 7 months [7].

A significant number of CN patients either present or subsequently develop bilateral Charcot foot. A weighted average of studies reporting bilateral involvement suggests 21% (range 9%–75%) of CN patients will present or subsequently develop Charcot foot [8–14]. Of those studies reporting subsequent development of CN, point estimates for bilateral involvement ranged from 2 to 3.3 years (range 1–6 years) after initial presentation. However, 21% of cases presented at baseline are with bilateral involvement [8, 10, 14]. This suggests a window of opportunity for the prevention of bilateral CN development. Certainly, a goal for identifying CN earlier is an important diagnostic pursuit, as well.

The role of thermometry in the detection of CN has been well described [8, 15, 16]. Armstrong and Lavery reported baseline infrared dermal thermometry results for 39 patients presenting with unilateral acute CN [15]. After 15 minutes' rest, they found an average 8.8 ± 2.3°F (~4.9 ± 1.3°C) difference in temperature compared to the contralateral joint of interest (JOI). In a separate study, the same team reported specific mean joint differences of 7.3°F (~4.1°C), 8.0°F (~4.4°C), and 8.8°F (~4.9°C) for the ankle Chopart and Lisfranc's joint, respectively [16]. Temperature differences correlate highly with radiographic changes [15] and with markers of bone turnover [17]. Offloading treatment should continue until temperature equilibration with the contralateral JOI [15] or within 2°C [18] is achieved. It is, however, unclear how temperature gradients changes are considered as a function of activity level. In this study, we examined temperature gradient changes of plantar temperature as a function of number of steps in patients with type 2 diabetes and peripheral neuropathy (DPN) including with and without acute CN.

## 2. Research Design and Methods

The study was conducted at a single academic medical center as part of a multinational collaborative study of lower extremity disease in diabetes. The study received ethical approval; participants were informed of the nature of the study and signed an informed consent form. Participants were included if they had type 2 diabetes diagnosed by their primary care physician and exhibited loss of protective sensation using 10-gram monofilament at 1–3 sites in the following locations: hallux, 1st, 3rd, and 5th metatarsal heads [19]. Patients with major foot amputation and inability to walk a distance of 100 m without assistance were excluded. The diagnosis of unilateral acute CN was made by a single clinician using previously described clinical criteria of swelling, redness, and local temperature gradient [20–22].

All participants walked for two predefined paths of 50 and 150 steps (total 200 steps). A validated wearable gait analysis technology (LEGSys, Biosensics LLC, MA, USA) [23–25] was used to assess gait and quantify the number of steps. All subjects were examined in prescribed footwear. In CN patients, this included nine with removable cast walkers, one with surgical sandal, and five with prescribed shoes. A thermal camera (Fluke Co., Model i25) was used to monitor plantar temperature at baseline after foot acclimatization and immediately after each walking trial. The subject was asked to sit in a podiatric examination chair with their legs parallel to the transverse plane and their shoes and socks removed for a 5-minute environment acclimatization period for baseline assessment. This was done to allow the subject's feet to equilibrate to room temperature. All subsequent thermal images (approximately at 50 steps and 200 steps) were taken with shoes and socks removed immediately after each walking trial. Due to the intermittent measurement at 50 steps, there was a slight delay (approximately 30 seconds) between continuation of the subsequent walking trial. We assumed that the change in plantar temperature is not rapid and thus this delay should have a negligible effect on assessing plantar temperature.

**Figure 1:**
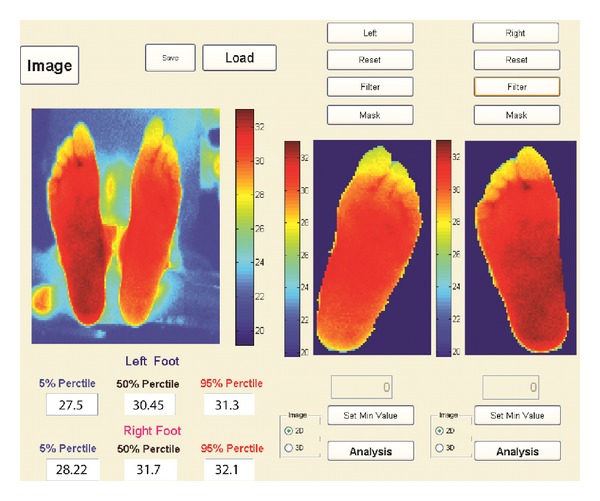
A purpose-designed image processing toolbox was developed using Matlab (version 7.4, The MathWorks, Inc., Natick, MA, USA), to isolate each foot from the thermal image and extract plantar temperature in three anatomical regions of foot including hind-, mid-, and forefoot.

A custom image processing toolbox (Figure 1) was designed using MATLAB version 7.4 (R2007z) (The MathWorks, Inc., Natick, MA, USA), to automatically isolate each foot from the thermal image using an edge detection algorithm. The toolbox also afforded manual enhancement and noise removal prior to the analysis. This is critical to accurately identify inflammatory hot spots and measure dynamic changes in plantar temperature. Using an automated masking algorithm, plantar temperature changes were measured in three independent anatomical regions (hind/mid/fore-foot). We estimated the 5th, 50th, and 95th temperature percentiles at each region. For the purpose of this study, only the 95th percentile value representing a hot spot was reported.

Paired sample Student's *t*-test was used to examine intra-subject PT differences between feet. A two-sample Student's *t*-test assuming equal variance was used between groups. ANOVA test (N-way analysis of variance) with linear model was used to examine the dependency of PT change on footwear type, gender, age, and active diabetic foot ulcer (DFU). Statistical significance was set at 0.05.

## 3. Results

**Figure 2:**
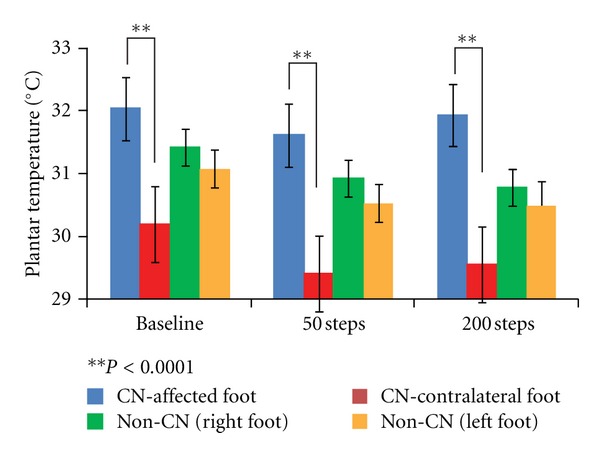
Plantar temperature in hot spot recognized in the mid-foot region.

**Figure 3:**
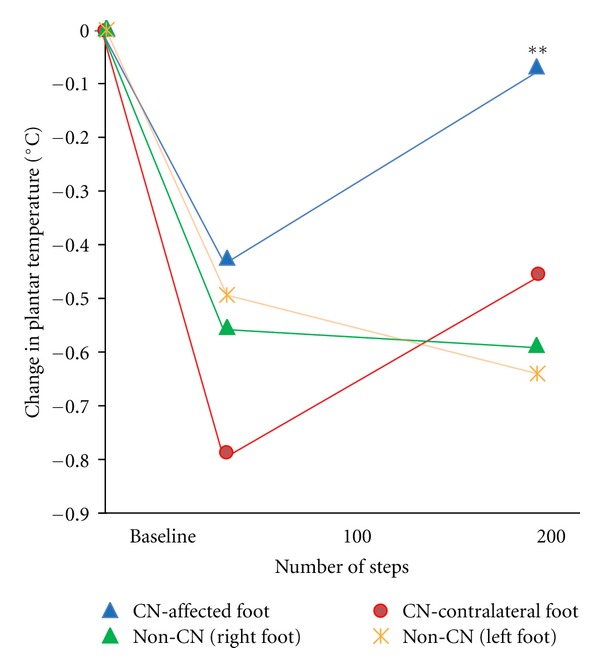
Change in plantar temperature as a function of walking steps for the hot spots recognized in the midfoot region.

Thirty-two eligible subjects (age: 56.6 ± 8.6 years, BMI = 30.3 ± 4.9 Kg/m^2^, 87% male) were recruited. Fifteen subjects were diagnosed with CN and 17 as non-CN. Eight CN and nine non-CN participants had DFU. Nine CN participants wore casts, one sandal, and five wore prescribed shoes. Eight non-CN participants wore their habitual shoes, six wore prescription shoes, and three wore surgical sandals. At baseline, CN demonstrated a significant 1.84 ± 1.3°C difference (*P* < 0.0001) between the feet at JOI and for all plantar regions (Figure 2). No significant difference was observed in non-CN (*P* > 0.3). Upon activity initiation, plantar temperature was reduced in all participants, but the drop for nonaffected foot as well as non-CN was significantly lower by a factor of 1.9 than the affected side (*P* = 0.04). Interestingly, plantar temperature in CN was sharply increased by prolong walking beyond of 50 steps with slope of 0.25 deg/100 steps for both feet, while no difference was observed in non-CN between 50 and 200 steps (*P* > 0.5, Figure 3). At 200 steps, plantar temperature on the CN side was still higher than the contralateral foot and non-CN (*P* < 0.0001).

Multivariable analysis suggested PT asymmetry measured at baseline as well as after each walking trial is independent of DFU, gender, age, and type of footwear (*P* > 0.1) but significantly dependent on presence of CN (*P* < 0.0001). Using PT gradient criteria based on the JOI, the effect size between CN and non-CN group was increased by 61% at 50 steps compared to baseline (*d* = 1.20 and *r* = 0.52 at baseline versus*d* = 1.94 and *r* = 0.70 at 50 steps).

## 4. Discussion

The current study reports a simple objective method to characterize asymmetry in plantar temperature as a function of graduated walking activity. This technique characterizes the PT hot spot (95th percentile) at each plantar region instead of manual comparison of plantar temperature between two feet.

We found that all participants experienced initial temperature decrease in both feet after 50 steps. But the slope of PT cooling to baseline was significantly slower in the affected foot. Consequently, the temperature difference between CN affected and contralateral foot is magnified after walking 50 steps (*d* = 1.20 and *r* = 0.52 at baseline versus*d* = 1.94 and *r* = 0.70 at 50 steps).

The initial drop in plantar temperature in early-walking steps may be due to regulation of microvascular flow in response to cyclic loading and relaxation. Silver-Thorn [26] by applying a cyclic loading and unloading to human healthy tissue demonstrated that skin perfusion is initially increased in response to early loading and dropped with further increases in pressure or prolong loading till reaching to a steady-state level (first pulse response). Then, again it is increased followed by decreasing to the initial value in response to unloading (second pulse response or hyperemia response), whereas little to no tissue reperfusion was observed during prolong relaxation period without cyclic stress. Therefore, cyclic activities like walking may actually increase the cumulative plantar skin perfusion as a function of time (or time integral) compared to prolong relaxing (e.g., sitting, lying, and offloading) or prolong loading (e.g., standing) conditions. Thus, this skin perfusion regulation in response to cyclic stress may explain the initial drop in plantar temperature in early steps compared to baseline (relaxation) for healthy skin when other factors contributing to increase in skin temperature (e.g., friction, metabolic cost, etc.) are still negligible. Considering that the most of walking episodes are short (often less than 50 steps per episode) [27], this regulation mechanism is of key importance in regulating foot temperature during activity of daily living. A failure in the above-explained skin perfusion regulation in response to cyclic plantar stress may explain the lack of drop in temperature in the CN-affected foot.

In non-CN, the temperature remained the same after continued activity from 50 steps up to 200 steps, but temperature was lower relative to the temperature at baseline. In CN, there was, however, a significant increase in temperature at 200 steps compared to 50, significantly higher than temperature difference between 50 and 200 steps in non-CN. These interesting findings merit further study as a potential stress test for prediction of unilateral or bilateral CN and subsequent ulcer development.

The sharp increase in plantar temperature in CN group after continued activity beyond 50 steps could be explained by a complex interplay between local metabolic status [28, 29], propensity for an ill-defined inflammatory overreaction [5, 29], perfusion status [30], the physical state of plantar tissue, and limited joint mobility which may increase skin friction or metabolic cost. There could be an empiric support for these findings from Johnson, who reported a sharp increase in plantar temperature in Charcot patients and postulated that it could be explained by hyperemia in Charcot foot [31]. According to Boulton et al., “It has been theorized that the site of pathology was within the arteriovenous shunts, which normally are under control of the sympathetic system. Loss of this function will result in blood being routed rapidly to the venous side of the capillary bed, increasing the local pO2, thereby decreasing the distal perfusion to the cells” [32]. These results also support that modulating duration of continuous steps and/or prolong standing during daily activity could be helpful for reducing the trauma in patients with CN or DFU [27, 33–35].

The temperature differences in our study differ from others at baseline [15, 16]. This could be due to the duration of acclimatization, use of a thermal imager as opposed to an infrared dermal thermometer, and aggregation of temperature into regions of the foot as opposed to manual point testing. Additionally, we have eliminated any bias towards absolute temperature measurements by using the 95th percentile values.

This study has few limitations. First, we were not able to control the stage of Charcot foot development. It is likely that some patients were in a coalescence phase. The magnitude of the differences between groups merit further investigation in stages 0 and 1 patients. It is entirely plausible that these patients are likely to have a higher thermal gradient. Second, we did not standardize the offloading footwear and, while our population was easily robust enough to assess temperature gradient, it was not sufficiently powered to perform a stratified analysis by stage and offloading footwear type. Third, due to limitations in technology, a short delay was required for assessing plantar temperature after each walking path. However, since the change in plantar temperature is not rapid, we assumed that the effect of this delay (approximately 30 seconds) for assessing change in plantar temperature as a function of walking is negligible. Another study should be addressed to validate this hypothesis.

The observed differential thermal response to walking initiation between Charcot and non-Charcot feet warrants future investigation to provide further insight into the correlation between activity dosing and thermal response. It may also lend valuable insight into identifying an “inflammatory trigger” that may ultimately provide an early-warning sign [36] or increased sensitivity for subsequent unilateral or bilateral CN development or clinical expression of foot ulcer. The importance of improved sensitivity and earlier diagnoses of CN was recently described by Wukich and colleagues. In their retrospective review of 22 CN patients, they emphasized the importance of identifying and aggressively treating stage 0 patients [6]. This was defined as patients with diabetes-related sensory neuropathy presenting following foot and ankle insult with local swelling, redness, and warmth and radiographic signs absent for fracture and normal alignment [37, 38]. The group that was identified and treated for 4 weeks developed significantly less complications (14%) versus the group that was identified and treated after 8 weeks (67%) [6].

In conclusion, the variability in thermal response to the initiation of walking between Charcot and non-Charcot feet warrants future investigation to potentially provide further insight into the correlation between thermal response and ulcer/Charcot development.

## References

[1] Gazis A, Pound N, Macfarlane R, Treece K, Game F, Jeffcoate W (2004). Mortality in patients with diabetic neuropathic osteoarthropathy (Charcot foot). *Diabetic Medicine*.

[2] Sohn MW, Lee TA, Stuck RM, Frykberg RG, Budiman-Mak E (2009). Mortality risk of charcot arthropathy compared with that of diabetic foot ulcer and diabetes alone. *Diabetes Care*.

[3] Sohn MW, Stuck RM, Pinzur M, Lee TA, Budiman-Mak E (2010). Lower-extremity amputation risk after charcot arthropathy and diabetic foot ulcer. *Diabetes Care*.

[4] Rogers LC, Frykberg RG, Armstrong DG (2011). The charcot foot in diabetes. *Diabetes Care*.

[5] Jeffcoate W (2008). The causes of the charcot syndrome. *Clinics in Podiatric Medicine and Surgery*.

[6] Wukich DK, Sung W, Wipf SAM, Armstrong DG (2011). The consequences of complacency: managing the effects of unrecognized charcot feet. *Diabetic Medicine*.

[7] Pakarinen TK, Laine HJ, Honkonen SE, Peltonen J, Oksala H, Lahtela J (2002). Charcot arthropathy of the diabetic foot. Current concepts and review of 36 cases. *Scandinavian Journal of Surgery*.

[8] Armstrong DG, Todd WF, Lavery LA, Harkless LB, Bushman TR (1997). The natural history of acute Charcot's arthropathy in a diabetic foot specialty clinic. *Diabetic Medicine*.

[9] Clohisy DR, Thompson RC (1988). Fractures associated with neuropathic arthropathy in adults who have juvenile-onset diabetes. *Journal of Bone and Joint Surgery A*.

[10] Fabrin J, Larsen K, Holstein PE (2000). Long-term follow-up in diabetic charcot feet with spontaneous onset. *Diabetes Care*.

[11] Herbst SA, Jones KB, Saltzman CL (2004). Pattern of diabetic neuropathic arthropathy associated with the peripheral bone mineral density. *Journal of Bone and Joint Surgery B*.

[12] Myerson MS, Henderson MR, Saxby T, Short KW (1994). Management of midfoot diabetic neuroarthropathy. *Foot and Ankle International*.

[13] Sinha S, Munichoodappa CS, Kozak GP (1972). Neuro-arthropathy (Charcot joints) in diabetes mellitus (clinical study of 101 cases). *Medicine*.

[14] Leung HB, Ho YC, Wong WC (2009). Charcot foot in a Hong Kong Chinese diabetic population. *Hong Kong Medical Journal*.

[15] Armstrong DG, Lavery LA (1997). Monitoring healing of acute Charcot’s arthropathy with infrared dermal thermometry. *Journal of Rehabilitation Research and Development*.

[16] Armstrong DG, Lavery LA, Liswood PJ, Todd WF, Tredwell JA (1997). Infrared dermal thermometry for the high-risk diabetic foot. *Physical Therapy*.

[17] Bem R, Jirkovská A, Dubský M (2010). Role of quantitative bone scanning in the assessment of bone turnover in patients with Charcot foot. *Diabetes Care*.

[18] Petrova NL, Edmonds ME (2008). Charcot neuro-osteoarthropathy—current standards. *Diabetes/Metabolism Research and Reviews*.

[19] Boulton AJ, Armstrong DG, Albert SF (2008). Comprehensive foot examination and risk assessment: a report of the task force of the foot care interest group of the American Diabetes Association, with endorsement by the American Association of Clinical Endocrinologists. *Diabetes Care*.

[20] Wukich DK, Sung W (2009). Charcot arthropathy of the foot and ankle: modern concepts and management review. *Journal of Diabetes and Its Complications*.

[21] Wukich DK, Sung W, Wipf SAM, Armstrong DG (2011). The consequences of complacency: managing the effects of unrecognized Charcot feet. *Diabetic Medicine*.

[22] Armstrong DG, Peters EJ (2002). Charcot’s arthropathy of the foot. *Journal of the American Podiatric Medical Association*.

[23] Najafi B, Helbostad JL, Moe-Nilssen R, Zijlstra W, Aminian K (2009). Does walking strategy in older people change as a function of walking distance?. *Gait and Posture*.

[24] Najafi B, Khan T, Wrobel J Laboratory in a box: wearable sensors and its advantages for gait analysis.

[25] Aminian K, Najafi B, Büla C, Leyvraz PF, Robert P (2002). Spatio-temporal parameters of gait measured by an ambulatory system using miniature gyroscopes. *Journal of Biomechanics*.

[26] Silver-Thorn MB (2002). Investigation of lower-limb tissue perfusion during loading. *Journal of Rehabilitation Research and Development*.

[27] Najafi B, Crews RT, Wrobel JS (2010). The importance of time spent standing for those at risk of diabetic foot ulceration. *Diabetes Care*.

[28] Frykberg RG, Belczyk R (2008). Epidemiology of the Charcot foot. *Clinics in Podiatric Medicine and Surgery*.

[29] Jeffcoate WJ, Game F, Cavanagh PR (2005). The role of proinflammatory cytokines in the cause of neuropathic osteoarthropathy (acute Charcot foot) in diabetes. *The Lancet*.

[30] Shapiro SA, Stansberry KB, Hill MA (1998). Normal blood flow response and vasomotion in the diabetic Charcot foot. *Journal of Diabetes and Its Complications*.

[31] Johnson JT (1967). Neuropathic fractures and joint injuries. Pathogenesis and rationale of prevention and treatment. *Journal of Bone and Joint Surgery A*.

[32] Boulton AJM, Scarpello JHB, Ward JD (1982). Venous oxygenation in the diabetic neuropathic foot: evidence of arteriovenous shunting?. *Diabetologia*.

[33] Armstrong DG, Boulton AJM (2001). Activity monitors: should we begin dosing activity as we dose a drug?. *Journal of the American Podiatric Medical Association*.

[34] Najafi B, Crews RT, Wrobel JS Incorporating standing time into physical activity assessment of patients at risk of diabetic foot ulceration.

[35] Wrobel JS, Najafi B (2010). Diabetic foot biomechanics and gait dysfunction. *Journal of Diabetes Science and Technology*.

[36] Wukich DK, Sung W, Wipf SAM, Armstrong DG (2011). The consequences of complacency: managing the effects of unrecognized Charcot feet. *Diabetic Medicine*.

[37] Yu GV, Hudson JR (2002). Evaluation and treatment of stage 0 Charcot’s neuroarthropathy of the foot and ankle. *Journal of the American Podiatric Medical Association*.

[38] Shibata T, Tada K, Hashizume C (1990). The results of athrodesis of the ankle for leprotic neuroarthropathy. *Journal of Bone and Joint Surgery A*.

